# A Hexahomotrioxacalix[3]arene-Based Ditopic Receptor for Alkylammonium Ions Controlled by Ag^+^ Ions

**DOI:** 10.3390/molecules23020467

**Published:** 2018-02-21

**Authors:** Xue-Kai Jiang, Yusuke Ikejiri, Chong Wu, Shofiur Rahman, Paris E. Georghiou, Xi Zeng, Mark R. J. Elsegood, Carl Redshaw, Simon J. Teat, Takehiko Yamato

**Affiliations:** 1 Department of of Applied Chemistry, Faculty of Science and Engineering, Saga University, Honjo-machi 1, Saga 840-8502 Japan; jxk1215@163.com (X.K.J.); k1xshamgod@gmail.com (Y.I.); wuchong214@163.com (C.W.); 2 Department of Chemistry, Memorial University of Newfoundland, St. John’s, NL A1B 3X7, Canada; mdrahman71@yahoo.com (S.R.); parisg@mun.ca (P.E.G.); 3 Key Laboratory of Macrocyclic and Supramolecular Chemistry of Guizhou Province, Guizhou University, Guiyang 550025, China; zengxi1962@163.com; 4 Chemistry Department, Loughborough University, Loughborough, LE11 3TU, UK; M.R.J.Elsegood@lboro.ac.uk; 5 Chemisty, School of Mathematics and Physical Sciences, The University of Hull, Cottingham Road, Hull, Yorkshire, HU6 7RX, UK; C.Redshaw@hull.ac.uk; 6 ALS, Berkeley Lab, 1 Cyclotron Road, Berkeley, CA 94720, USA; sjteat@lbl.gov

**Keywords:** hexahomotrioxacalix[3]arene, alkylammonium ions, metal ions, ditopic receptor, allosteric effects

## Abstract

A receptor *cone*-**1** based on a hexahomotrioxacalix[3]arene bearing three pyridyl groups was successfully synthesized, which has a *C*_3_-symmetric conformation and is capable of binding alkylammonium and metal ions simultaneously in a cooperative fashion. It can bind alkylammonium ions through the π-cavity formed by three aryl rings. This behaviour is consistent with the cone-in/cone-out conformational rearrangement needed to reorganize the cavity for *endo*-complexation. As a *C*_3_-symmetrical pyridyl-substituted calixarene, receptor *cone*-**1** can also bind an Ag^+^ ion, and the nitrogen atoms are turned towards the inside of the cavity and interact with Ag^+^. After complexation of tris(2-pyridylamide) derivative receptor *cone*-**1** with Ag^+^, the original *C*_3_-symmetry was retained and higher complexation selectivity for *n*-BuNH_3_^+^ versus *t*-BuNH_3_^+^ was observed. Thus, it is believed that this receptor will have a role to play in the sensing, detection, and recognition of Ag^+^ and *n*-BuNH_3_^+^ ions.

## 1. Introduction

Over the past few decades, the development of artificial receptors for ion recognition, complexation, and transportation has proven to be an important topic in both environmental and supramolecular chemistry [1,2,3,4]. Given their ready availability, and their unique conformational and cavity-containing structures, together with their versatile molecular recognition properties, calixarenes have attracted a great deal of attention during the past several decades, and, indeed, calixarene chemistry has become an indispensable part of supramolecular science [5,6,7,8,9]. Calix[*n*]arenes can provide useful building blocks for host-guest type receptors by appropriate modification. For example, calix[4]arene derivatives incorporating crown ethers, amides, esters, and carboxylic acid groups have been shown to selectively extract metal ions [10]. Hexahomotrioxacalix[3]arenes, which are structurally related to both calixarenes and to crown ethers, have a three dimensional cavity with a potentially *C*_3_-symmetric structure, and have been shown to be useful ligands for metal cations [11,12,13,14], ammonium cations [15,16], and fullerene derivatives [17,18].

Cation recognition by artificial receptors has attracted increasing attention due to the important roles played by ions in both environmental and biological systems [19,20]. All the known modes of cation binding by native and functionalized calixarenes exploit cation-π induced dipoles, or electrostatic interactions [21]. The most important calixarene-based cation receptors are obtained by the introduction of chelating units at the lower rather than at the upper rim. For example, calixarenes fully functionalized at the lower rim with ether groups show an affinity for alkali metal ions [22,23,24,25]. Shinkai et al. reported a series of calix[4]arene-crown-4 derivatives, among which a *partial-cone* derivative exhibits an exceptional Na^+^/K^+^ selectivity, as determined by ion-selective electrodes (ISEs) [23]. We also have developed a series of triazole-derived chemosensors for selective binding of heavy metal ions based on hexahomotrioxacalix[3]arene and thiacalix[4]arene scaffolds [26,27,28]. For example, chemosensors derived from hexahomotrioxacalix[3]arene appended at the lower rim with pyrenyl groups via triazole linkers exhibited a highly selective affinity for the Pb^2+^ cation through enhancement of the monomer emission of the pyrene moiety in both organic and organic-aqueous solution [26].

On the other hand, hexahomotrioxacalix[3]arene derivatives that have *C*_3_ symmetry can also selectively bind ammonium ions, which play an important role in both chemistry and biology. The formation of intra-cavity *endo*-complexes of the resulting alkylammonium ions have been reported. Tripodal NH^+^···O interactions with the phenolic oxygen atoms and C–H···π interactions stabilized the complex [1,2,3,4,11]. Recently, our group reported the construction of *C*_3_ symmetrically functionalized hexahomotrioxacalix[3]arenes, which selectively recognized primary alkylammonium ions [29,30].

It is against this background that we describe herein the design, synthesis, and binding properties of receptor *cone*-**1**, based on hexahomotrioxacalix[3]arene, which recognizes alkylammonium cations and Ag^+^ with high association constants.

## 2. Results and Discussion

**Scheme 1:**
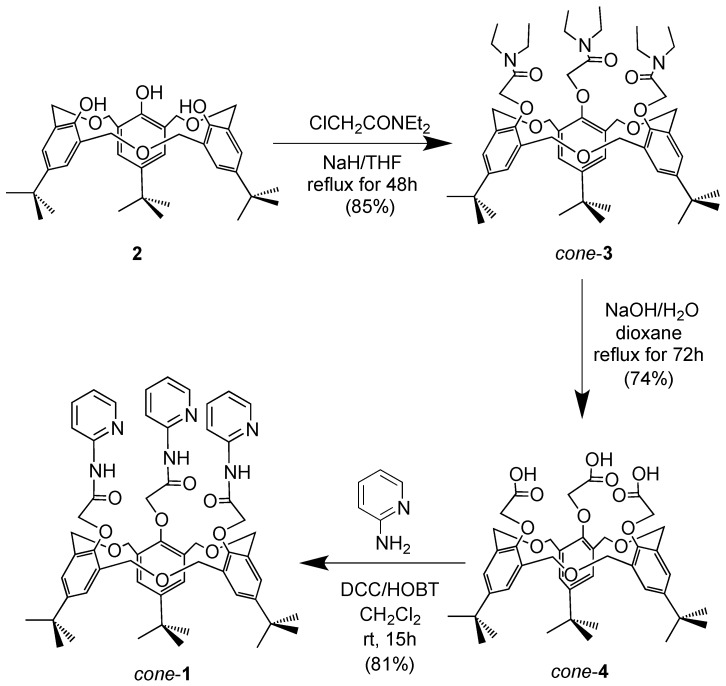
Synthesis of receptor *cone*-**1**.

*Cone*-**1,** which is based on a hexahomotrioxacalix[3]arene, was synthesized by the method shown in Scheme 1. *Cone*-**3** was prepared by *O*-alkylation of hexahomotrioxacalix[3]arene **2** with *N,N*-diethylchloroacetamide in the presence of NaH in refluxing THF according to the reported procedure in 85% yield [31,32]. *Cone*-**4** was synthesized in 74% yield by hydrolyzing *cone*-**3** in a refluxing mixture of NaOH/H_2_O/dioxane solution. The *cone*-hexahomotrioxacalix[3]arene triamide derivative (*cone*-**1**) was prepared in 81% yield by a condensation reaction between *cone*-**4** and 2-aminopyridine in the presence of DCC (dicyclohexylcarbodiimide) and HOBt (1-hydroxybenzotriazole) at room temperature for 15 h in CH_2_Cl_2_.

In agreement with its *C*_3_-symmetric *cone*-in conformation, receptor *cone*-**1** displays the following characteristic ^1^H and ^13^C-NMR spectroscopic features: (1) the *cone*-**1** conformation is firmly established by the presence of the bridging methylene protons that have a Δδ separation between H_ax_ and H_eq_ of Δδ 0.41 ppm in the ^1^H-NMR spectrum (CDCl_3_). In the calix[4]arenes, the Δδ values of the ArCH_2_Ar methylene protons have been correlated with the orientation of the adjacent aromatic rings [33,34] and similar findings were previously observed with hexahomotrioxacalix[3]arenes [12,13]. (2) The ^13^C-NMR spectrum of receptor *cone*-**1** in CDCl_3_ exhibited two peaks at δ 31 and 34 ppm for the *tert*-butyl carbons and a single peak at δ 69 ppm for the -O*C*H_2_- bridge linker carbons [35]. (3) The ^1^H-NMR spectrum of receptor *cone*-**1** in CDCl_3_ revealed a single peak at δ 1.15 ppm for the *tert*-butyl protons and a single peak at δ 6.99 ppm for the aromatic protons, which was in agreement with a *C*_3v_-symmetric structure [36].

**Figure 1:**
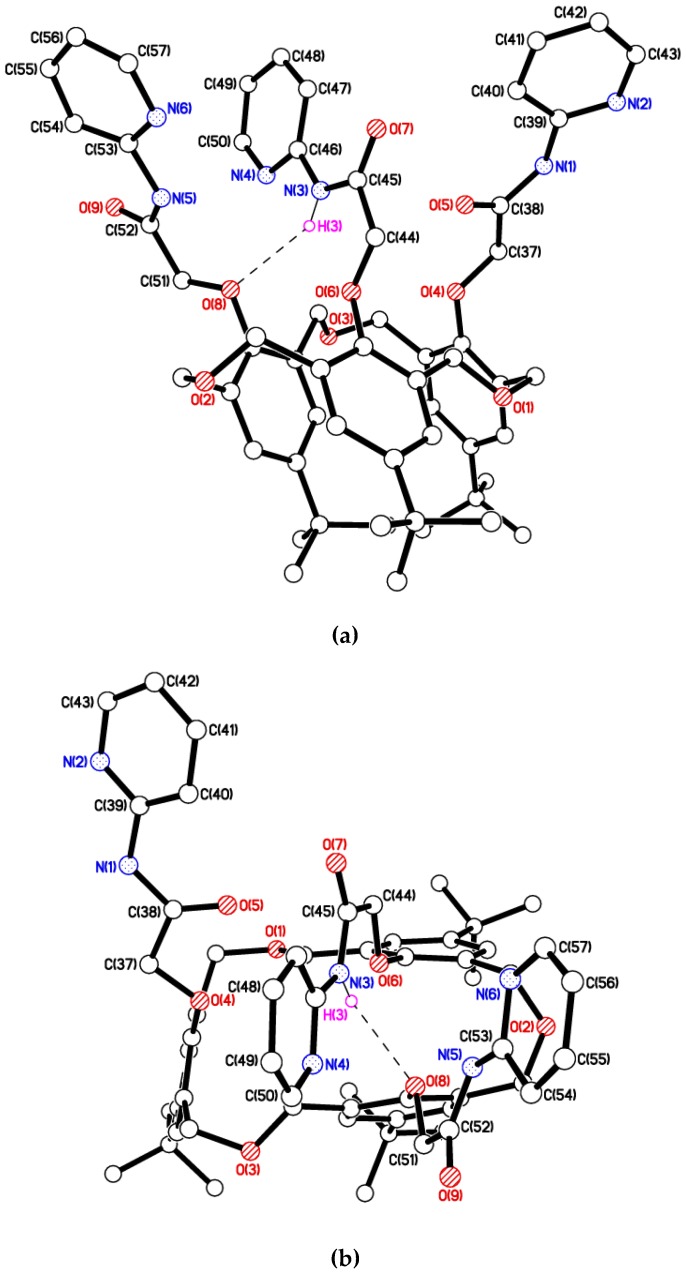
Side-view (**a**) and top-view (**b**) of the X-ray structure of *cone*-**1**. H-bonds shown as dashed lines; H(3)···O(8) = 2.29(2) Å, ∠N(3)–H(3)···O(8) = 147(2). Solvent molecules of crystallization and H atoms not involved in H-bonding omitted for clarity.

Single crystal X-ray diffraction studies of *cone*-**1** shows a rather deformed *cone* conformation (Figure 1). *Cone*-**1** crystallises as solvates incorporating either three methanol molecules, and one water molecule or with 2½ methanol molecules. Within each calixarene is a single N–H···O H-bond between a secondary amine moiety and a single ether oxygen atom. See experimental section and Table S1 for crystal data.

In *cone*-**1**∙3 MeOH∙H_2_O the calixarene molecule adopts a collapsed, or squashed, conformation to facilitate this H-bond and a π···π interaction between the arene rings C(13)–C(18) and C(25)–C(30), at a distance of 3.87 Å. The pyridyl groups C(46)–N(4) and C(53)–N(6) are aligned face-to-face but at a distance of 4.80 Å, which is too far for π···π stacking to be adopted. The structure of *cone*-**1**∙2.5 MeOH is very similar and is isomorphous (see Figures S7 and S8).

**Figure 2:**
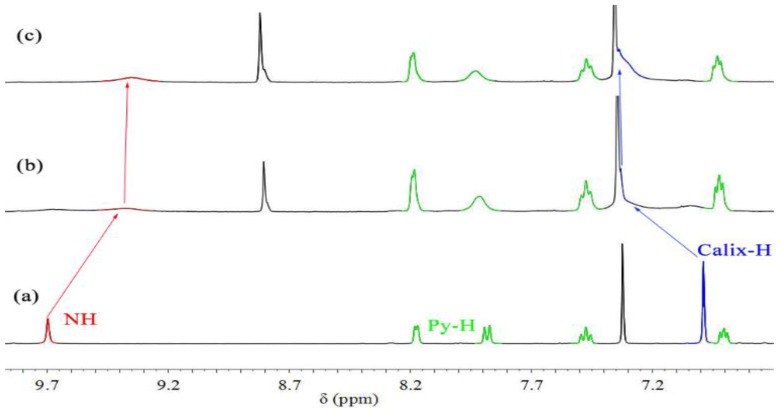
Partial ^1^H-NMR spectra of receptor *cone*-**1** (5.0 mM) in CDCl_3_/CD_3_CN (10:1, *v*/*v*); (**a**) *cone*-**1**; (**b**) *cone*-**1** ⊃ *n*-BuNH_3_^+^ (0.6 equiv.); (**c**) *cone*-**1** ⊃ *n*-BuNH_3_^+^ (1.0 equiv.).

In order to investigate the ionophoric affinities of the receptor *cone*-**1** for alkylammonium ions, the binding properties of receptor *cone*-**1** as a ditopic receptor were investigated by means of ^1^H-NMR spectroscopic titration experiments, using *n*-butylammonium and *t*-butylammonium picrates as the substrates. Binding studies were carried out by adding increasing amounts of the appropriate salt to a 5.0 mM solution of receptor *cone*-**1** in CDCl_3__/_CD_3_CN co-solvent, so as to reach a 1:1 host/guest ratio. In the case of *n*-BuNH_3_^+^, the proton signals of receptor *cone*-**1** were observed for both the complex and the free host, as shown in Figure 2. Upon increasing the amount of alkylammonium ions, the signals for receptor *cone*-**1** decreased, and, finally, only signals for the complex were observed. Host-guest complexation/decomplexation was judged to be slow on the NMR time scale on the basis of preliminary titration experiments, which, in the presence of greater host than guest concentrations, revealed broadness for the receptor *cone*-**1** peaks. The peak assigned to the aromatic hydrogen atoms of the hexahomotrioxacalix[3]arene unit underwent a significant down-field complexation-induced shift (CIS) (from δ 6.99 to 7.31 ppm), indicating that the cavity was ‘opening-up’ to make room for the incoming cationic guest.

**Figure 3:**
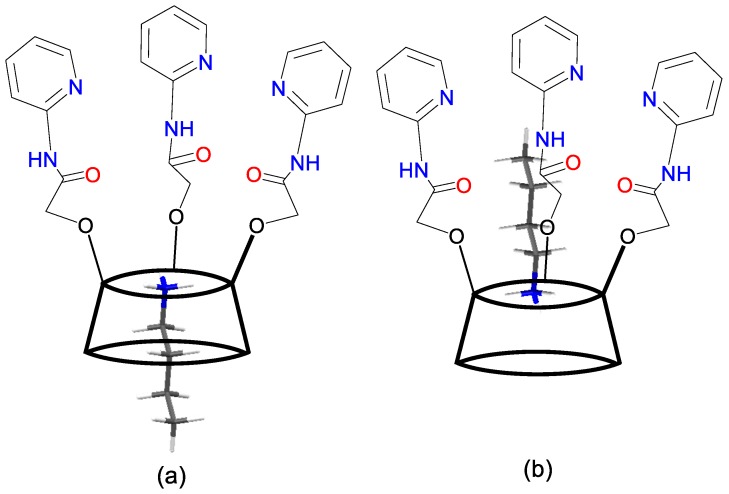
Two possible interactions between receptor *cone*-**1** and *n*-BuNH_3_^+^.

This behaviour is consistent with a *cone-in-cone-out* conformational rearrangement needed to reorganize the cavity upon *endo*-complexation. On the other hand, there are two modes for receptor *cone*-**1** to bind with *n*-butylammonium ions, i.e., either at the lower rim through the substituent moieties, or at the upper rim through the π-cavity formed by the three aromatic rings (Figure 3). The presence of the target *n*-BuNH_3_^+^ cation was evident from the appearance of high-field resonances for the included *n*-butyl chain as presented in Table 1 [37,38]. It suggests that the alkyl protons of the *n*-BuNH_3_^+^ ion reside inside the cavity of receptor *cone*-**1** and are subjected to a shielding effect from the phenyl rings (Figure 3a) [39,40,41,42]. This evidence suggests the binding occurs through the π-cavity formed by the three aromatic rings of the hexahomotrioxacalix[3]arene unit. This binding is attributed to the π-effect of the aromatic rings and is favoured, since both the host and the guest molecules have *C*_3_-symmetric conformations.

Similar ^1^H-NMR spectroscopic titration experiments were conducted to assess the effect of *t*-BuNH_3_^+^, and the spectral changes upon complexation are shown in Table 1 and Figure S3. The association constants of receptor *cone*-**1** with alkylammonium ions were determined and are presented in Table 2. It can be seen that the association constants were affected by the sizes of the respective alkyl groups. Thus, receptor *cone*-**1** binds more readily to the linear alkyl chain ammonium ion than to the branched chain ammonium ion. These findings might be attributable to the greater ease of entering the cavity for the linear alkyl chain analogs, as well as the slower decomplexation rate of the ammonium ions.

**Figure 4:**
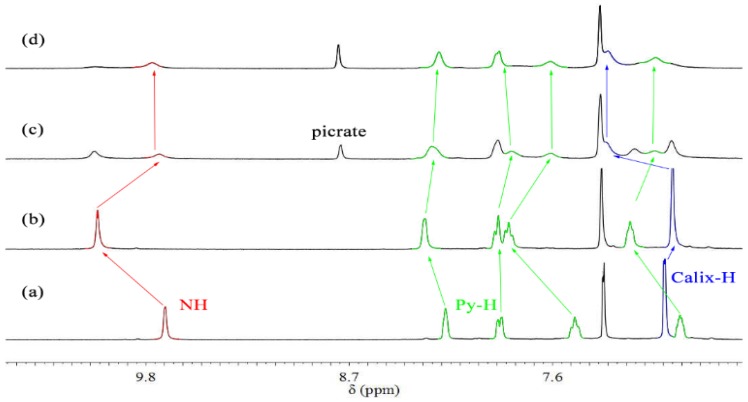
Partial ^1^H-NMR spectra of receptor *cone*-**1** (CDCl_3_/CD_3_CN (10:1, *v*/*v*), 5.0 mM); (**a**) *cone*-**1**; (**b**) *cone*-**1** ⊃ Ag^+^ (1.0 equiv.); (**c**) *cone*-**1** ⊃ Ag^+^ (1.0 equiv.) ⊃ *n*-BuNH_3_^+^ (0.5 equiv.); (**d**) *cone*-**1** ⊃ Ag^+^ (1.0 equiv.) ⊃ *n*-BuNH_3_^+^ (1.0 equiv.).

On the other hand, host receptor *cone*-**1** was recently reported by us to also have excellent affinity and high selectivity for Ag^+^, Cu^2+^, and Al^3+^ ions during extraction studies [31,42]. In particular, we reported that receptor *cone*-**1** exhibited higher extractability for Ag^+^ (76.9%), with the Ag^+^ being complexed by the three pyridine groups via N–Ag^+^ interactions. Therefore, in order to determine optimized conditions for recognition and selectivity of the ammonium ions, the effects on ^1^H-NMR spectroscopic titrations of *cone*-**1**·Ag^+^ toward ammonium ions were studied in CDCl_3_/CD_3_CN (10:1, *v*/*v*). In the case of *n*-BuNH_3_^+^, partial ^1^H-NMR spectra (Figure 4) revealed that the proton resonances of the *n*-butyl chain were shifted upfield and the proton resonances of the aromatic hydrogen atoms of the hexahomotrioxacalix[3]arene units were shifted downfield by 0.11 ppm (from 6.99 to 7.29 ppm). These findings imply that Ag^+^ was encapsulated into the cavity formed by the pyridine rings, and the receptor *cone*-**1** ‘stands up’ when *n*-BuNH_3_^+^ is included, because *n*-BuNH_3_^+^ enters into the cavity formed by the three aromatic rings.

Receptor *cone*-**1** is capable of binding both Ag^+^ and *n*-BuNH_3_^+^ simultaneously in a cooperative fashion. Thus, the potential to act as a ditopic receptor to complex with these two cations will be of great interest in coordination chemistry. Interestingly, similar ^1^H-NMR spectroscopic titration experiments were conducted to assess the effect of *t*-BuNH_3_^+^, but no significant changes in the resulting ^1^H-NMR spectra were observed. Thus, it is evident that receptor *cone*-**1** has lost the capacity to bind *t*-BuNH_3_^+^ when in the presence of Ag^+^ ion.

When the order of addition of Ag^+^ and alkylammonium ions was changed, in the case of *n*-BuNH_3_^+^, the resulting ^1^H-NMR spectrum is identical to that obtained in the previous section (Figure S4). Furthermore, in the case of *t*-BuNH_3_^+^, the resulting final ^1^H-NMR spectrum is the same as that for the complex *cone*-**1**·Ag^+^. These findings indicate that receptor *cone*-**1** does not selectively recognize alkylammonium ions efficiently in the absence of Ag^+^ ion. Interestingly, when Ag^+^ ion was added to the present system, Ag^+^ provided an excellent pathway of organizing alkylammonium ions binding groups for optimal host-guest interactions by adjusting the size of the cavity of receptor *cone*-**1**. The association constants of receptor *cone*-**1** with the alkylammonium ions in the absence of and in the presence of Ag^+^ as calculated from the chemical shift changes of the NH protons are summarized in Table 2. Interestingly, the association constant *K*_a_ for the complexation of receptor *cone*-**1** with *n*-BuNH_3_^+^ (*K*_a_ = 2680 ± 155) is much larger than that when using *t*-BuNH_3_^+^ (*K*_a_ = 360 ± 20). Thus, in the presence of Ag^+^, the complexation with *n*-BuNH_3_^+^ increases compared with *t*-BuNH_3_^+^, presumably due to the different cavity size of receptor *cone*-**1**.

**Figure 5:**
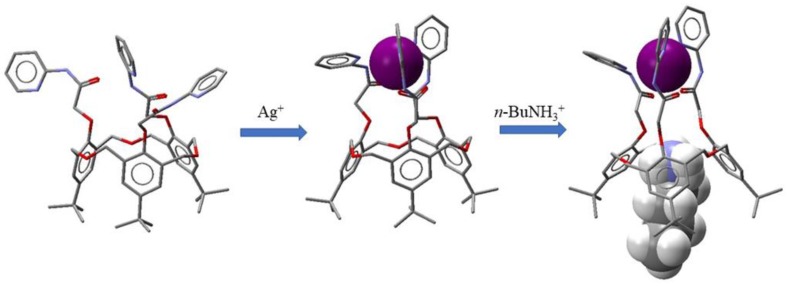
Geometry-optimized (PBE0/LANL2DZ) structures of *cone*-**1** and its complex with Ag^+^ and *n*-BuNH_3_^+^. Left: The free *cone*-**1**, Middle: 1:1 *cone*-**1** ⊃ Ag^+^ complex, and Right*: n*-BuNH_3_^+^ ⊂ [*cone*-**1** ⊃ Ag^+^] complex. Colour code: carbon = black grey, oxygen atom = red, nitrogen = blue, nitrogen (*n*- BuNH_3_^+^) = magenta and Ag^+^ = purple. Hydrogen atoms on the host molecule have been removed for clarity.

**Figure 6:**
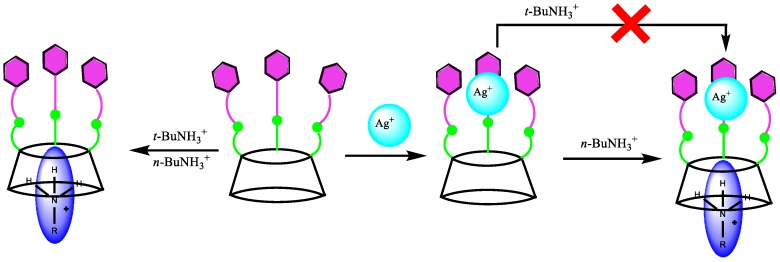
A plausible binding model. The green circles represent the -CONH-, and the hexagons (purple color) represent the pyridyl groups.

The geometries of the molecular structures were optimized with the PBE0 functional theory with the LANL2DZ basis set. The DFT level of theory used the hybrid Perdew-Burke-Ernzerhof parameter free-exchange correlation functional PBE0 (PBE1PBE in the Gaussian realization) [43,44] with the Hay and Wadt effective core potential LANL2DZ basis set [45,46,47]. The starting structure was generated using SpartanPro10 with the MMFF94 method [48]. The generated structures were then imported into Gaussian-09 Revision D.01 (Wallingford, CT, USA) [49]. and were geometry-optimized in the gas phase. The calculated binding or interaction energies (IE) for *cone*-**1** ⊃ *n*-BuNH_3_^+^, *cone*-**1** ⊃ *t*-BuNH_3_^+^, *cone*-**1** ⊃ Ag^+^ , and *n*-BuNH_3_^+^ ⊃ [*cone*-**1** ⊃ Ag^+^] are −298.8 kJ mol^−1^; –268.3 kJ·mol^−1^; –457.1 kJ·mol^−1^, and –525.8 kJ·mol^−1^, respectively, and were in agreement with the trend for the observed complexation data obtained by the ^1^H-NMR spectroscopic titration experiments (Figure 5). A conceptualization of complexation by the receptor *cone*-**1** is shown in Figure 6.

## 3. Experimental Section

### 3.1. General

All melting points were determined using a Yanagimoto MP-S1melting point apparatus (Kyoto, Japan). ^1^H-NMR spectra were recorded at 300 MHz with a Nippon Denshi JEOL FT-300 NMR spectrometer (Tokyo, Japan) with SiMe_4_ as an internal reference; *J* values are given in Hz. IR spectra were measured as KBr pellets on a Nippon Denshi JIR-AQ2OM spectrophotometer. UV-vis. spectra were measured with a Shimadzu 240 spectrophotometer (Tokyo, Japan). Mass spectra were obtained on a Nippon Denshi JMS-01SG-2 mass spectrometer at an ionization energy of 70 eV using a direct inlet system through GLC. Elemental analyses were performed with a Yanaco MT-5 (Kyoto, Japan).

### 3.2. Materials

Synthesis of *cone*-7,15,23-tri-*tert*-butyl-25,26,27-tris[(*N,N*-diethylaminocarbonyl)methoxy]-2,3,10,11,18,19-hexahomo-3,11,19-trioxacalix[3]arene (*cone*-**3**) was carried out according to the previously reported procedure [31].

### 3.3. Preparation of cone-4

A mixture of *cone*-**3** (500 mg, 0.55 mmol) and 15 mL of an aqueous 1.0 M NaOH solution in dioxane (15 mL) was heated at reflux for 72 h under N_2_. After cooling the reaction mixture to room temperature, the solution was acidified with 1M HCl (pH 1–2) and extracted three times (30 mL × 3) with ethyl acetate. The organic layer was separated, dried (MgSO_4_), and the solvent was evaporated. The residue was dried to afford *cone*-**4,** which was washed with methanol to afford the desired product *cone*-**4** as a white solid (320 mg, 74%), m.p. 227–229 °C (lit. [42], m.p. 227–229 °C).

### 3.4. Preparation of cone-1

A solution of *cone*-**4** (100 mg, 0.13 mmol), 2-aminopyridine (120 mg, 1.11 mmol), and HOBt (75 mg, 0.49 mmol) in dry CH_2_Cl_2_ (15 mL) was cooled in an ice bath. DCC (560 mg, 2.71 mmol) and dry CH_2_Cl_2_ (5 mL) were added, and the mixture was stirred for an additional 15 h at r.t., and then the solvent was evaporated. The residue was extracted three times (3 × 30 mL) with ethyl acetate. The organic layer was separated, dried (MgSO_4_), and the solvent was evaporated. The residue was dried to afford **1** as a colourless oil, which was recrystallized from CHCl_3_–hexane (1:3 *v*/*v*) to afford the desired product *cone*-**1** (85 mg, 81%) as colourless prisms. M.p. 217–218 °C. IR: ν_max_ (KBr)/cm^−^^1^ 3345, 3015, 2915, 2867, 1758, 1483, 1456, 1363, 1234, 1199, 1094 and 1058. ^1^H-NMR (300 MHz, CDCl_3_): δ_H_ = 1.15 (s, 27H, *t*-Bu-H), 4.49 (s, 6H, –CH_2_), 4.53 (d, *J* = 11 Hz, 6H, –OCH_2_), 4.94 (d, *J* = 11 Hz, 6H, –OCH_2_), 6.89 (m, 3H, Py–H), 6.99 (s, 6H, Calix-H), 7.47 (m, 3H, Py–H), 7.87 (d, 3H, *J* = 8.0 Hz, Py–H), 8.17 (d, *J =* 8.0 Hz, 3H, Py–H) and 9.69 (s, 3H, NH) ppm. ^13^C-NMR (100 MHz, CDCl_3_): δ_C_ = 31.42, 34.26, 69.65, 73.16, 113.92, 119.65, 126.94, 130.54, 137.58, 147.05, 147.74, 150.74, 152.58 and 167.45 ppm. FABMS: *m/z* calcd for C_57_H_66_N_6_O_9_ 978.17 [M^+^]; found 978.45 [M^+^]. C_57_H_66_N_6_O_9_ (978.17): calcd C 69.92, H 6.79; found: C 69.74, H 6.93.

### 3.5. ^1^H-NMR Spectroscopic Complexation Experiments

To a CDCl_3_/CD_3_CN solution (10:1, *v*/*v*) of *cone*-**1** (4 × 10^−3^ M) in an NMR tube was added 1.0 equivalent of related metal perchlorates. The spectrum was recorded after addition, and the temperature of the NMR probe was kept constant at 27 °C.

The ^1^H-NMR spectroscopic data of the host molecules of the respective complexes only are given below: The ^1^H-NMR data of the most representative complexes is given below:

**Receptor *cone*-1 ⊃ *n*-BuNH_3_^+^**: δ_H_ (CDCl_3_/CD_3_CN, 10:1): 1.24 (s, 27H, *t*-Bu–*H*), 4.36 (bs, 6H, –O*CH_2_*), 4.94 (bs, 6H, –*CH_2_*), 5.32 (bs, 6H, –O*CH_2_*), 6.91 (m, 3H, Py–*H*), 7.31 (s, 6H, Calix–*H*), 7.45 (m, 3H, Py–*H*), 7.91 (m, 3H, Py–*H*), 8.17 (d, 3H, *J* = 3.0 Hz, Py–*H*) and 9.35 (s, 3H, *NH*) ppm.

**Receptor *cone*-1 ⊃ *t*-BuNH_3_^+^**: δ_H_ (CDCl_3_/CD_3_CN, 10:1): 1.23 (s, 27H, *t*-Bu–*H*), 4.49 (bs, 6H, –O*CH_2_*), 5.11 (bs, 6H, –*CH_2_*), 5.40 (m, 6H, –O*CH_2_*), 6.93 (t, *J* = 6.6 Hz, 3H, Py–*H*), 7.29 (s, 6H, Calix–*H*), 7.48 (m, 3H, Py–*H*), 7.91 (m, 3H, Py–*H*), 8.17 (d, 3H, *J* = 3.0 Hz, Py–*H*) and 9.35 (s, 3H, *NH*) ppm.

**Receptor *cone*-1 ⊃ Ag+:** δ_H_ (CDCl_3_/CD_3_CN, 10:1): 1.01 (s, 27H, *t*-Bu–*H*), 4.20 (d, *J* = 12.0 Hz, 6H, –O*CH_2_*), 4.28 (s, 6H, –*CH_2_*), 4.84 (d, 6H, *J* = 12.0 Hz, –O*CH_2_*), 6.81 (s, 6H, Calix–*H*), 7.07 (m, 3H, Py–*H*), 7.76 (m, 6H, Py–*H*), 8.17 (d, 3H, *J* = 3.8 Hz, Py–*H*) and 9.96 (s, 3H, *NH*) ppm.

**Receptor *cone*-1 ⊃ Ag+ ⊃*n*-BuNH_3_^+^**: δ_H_ (CDCl_3_/CD_3_CN, 10:1): 1.26 (s, 27H, *t*-Bu–*H*), 4.35 (d, 6H, *J* = 9 Hz, –O*CH_2_*), 4.99 (d, *J* = 9 Hz, 6H, –*CH_2_*), 5.32 (b, 6H, –O*CH_2_*), 7.05 (b, 3H, Py–*H*), 7.29 (s, 6H, Calix–*H*), 7.62 (m, 3H, Py–*H*), 7.92 (d, 3H, *J* = 7.5 Py–*H*), 8.19 (d, 3H, *J* = 4.5 Hz, Py–*H*) and 9.80 (s, 3H, *NH*) ppm.

### 3.6. Single-Crystal X-Ray Diffraction Measurements for cone-**1**

A suitable single crystal (size *ca*. 0.30 × 0.17 × 0.03 mm^3^) was selected and mounted on a Bruker APEX 2 CCD diffractometer (Billerica, MA, USA) equipped with synchrotron radiation (λ = 0.7805 Å) at ALS Station 11.3.1 [49]. Data were corrected for Lorentz and polarisation effects and for absorption [50]. Crystal data for *cone*-**1**·3 (CH_4_O)·H_2_O: C_57_H_66_N_6_O_9_·3(CH_4_O)·H_2_O, M = 1093.30, monoclinic, *C*2/*c*, *a* = 26.1641(11), *b* = 15.4995(6), *c* = 28.5153(11) Å, *β* = 94.063(3)°, *V* = 11534.8(8) Å^3^, *Z* = 8, *μ* = 0.09 mm^−1^, 113193 reflections measured, 22109 unique, *R*_int_ = 0.074, *R*1[*F*^2^ > 2σ(*F*^2^)] = 0.067, *wR*2 (all data) = 0.205 [51]. The *o*-pyridyl group C(53) > N(6) and the *tert*-butyl group C(7) > C(10) were modelled as fully disordered over two sets of positions. The solvent molecules of crystallisation were modelled using the Platon Squeeze procedure due to substantial disorder [52]. Crystal data for *cone*-**1**·2.5(CH_4_O): C_57_H_66_N_6_O_9_·2.5(CH_4_O, M = 1059.26, monoclinic, *C*2/*c*, *a* = 26.308(2), *b* = 15.6159(14), *c* = 28.644(3) Å, *β* = 94.1811(15)°, *V* = 11736.3(18) Å^3^, *Z* = 8, *μ* = 0.08 mm^−1^, 67896 reflections measured, 17827 unique, *R*_int_ = 0.053, *R*1[*F*^2^ > 2σ(*F*^2^)] = 0.059, *wR*2 (all data) = 0.178 [50]. The *o*-pyridyl group C(51) > N(6) and the *tert*-butyl group C(27) > C(29) were modelled as fully disordered over two sets of positions. The solvent molecules of crystallisation were modelled using the Platon Squeeze procedure due to substantial disorder except for atom O(10) due to its proximity to the disordered ring [52]. Further details of the crystal parameters, data collection conditions, and refinement parameters are summarized in Table S2. CCDC 1500977 & 1817851 contain the supplementary crystallographic data for this paper. These data can be obtained free of charge from The Cambridge Crystallographic Data Centre via www.ccdc.cam.ac.uk/data_request/cif.

## 4. Conclusions

In conclusion, a novel receptor *cone*-**1** based on hexahomotrioxacalix[3]arene was successfully synthesized, and was found to possess a *C*_3_-symmetric conformation. It can bind alkylammonium ions through the π-cavity formed by three aryl rings. As a *C*_3_-symmetrical pyridyl-substituted calixarene, ionophore receptor *cone*-**1** can bind an Ag^+^ ion, and after complexation of the tris(2-pyridylamide) derivative receptor *cone*-**1** with Ag^+^, the original *C*_3_-symmetry has been retained, and the complex showed high selectivity for *n*-BuNH_3_^+^, but not for *t*-BuNH_3_^+^. Thus, it is believed that this receptor will have a role to play in the sensing, detection, and recognition of Ag^+^ and *n*-BuNH_3_^+^ ions simultaneously.

## Figures and Tables

**Table 1:** Selected proton chemical shifts (δ, ppm) (300 MHz, CDCl_3_:CD_3_CN, 10:1 *v*/*v*, 27 °C) of guest.^a^

		Chemical Shift of Proton			
		CH_3_	CH_3_CH_2_	CH_3_CH_2_CH_2_	CH_2_
	Free	0.94	1.40	1.67	3.02
*n*-BuNH_3_^+^	Complex	0.88	0.30	−0.33	0.30
	Δδ	−0.06	−1.10	−2.00	−2.72
	Free	1.40			
*t*-BuNH_3_^+^	Complex	−0.34			
	Δδ	−1.74			

^a^ chemical shift (δ): ppm. Δδ = δ_complex_ − δ_free_.

**Table 2:** Association constants *K*_a_ (M^−1^) of host receptor *cone*-**1** with alkylammonium ions, with and without Ag^+^ picrate salt. ^a,b^

	*K*_a_ (M^−1^)
*n*-BuNH_3_^+^	2680 ± 155
*n*-BuNH_3_^+^ ⊂ [*cone*-**1** ⊃ Ag^+^]	3270 ± 190
*t*-BuNH_3_^+^	360 ± 20
*t*-BuNH_3_^+^ ⊂ [*cone*-**1** ⊃ Ag^+^]	No complexation

^a^ Measured in CDCl_3_/CD_3_CN (10:1, *v*/*v*) at 27 ^°^C by the ^1^H-NMR titration method, noting the chemical shift change of the NH proton; host concentration was 5.0 mM. ^b^ Alkylammonium cations were used as their picrate salts.

## Supplementary Materials

The following are available online, Figure S1: ^1^H-NMR spectrum of the synthesized receptor *cone*-**1**, Figure S2: 13C-NMR spectrum of the synthesized receptor *cone*-**1**, Figure S3: ^1^H-NMR titration experiments for *cone*-**1** with *t*-BuNH_3_^+^ and Ag^+^, Figure S4: ^1^H-NMR titration experiments for *cone*-**1** with *n*-BuNH_3_^+^ and Ag+, Figure S5: molar ratio of Ag^+^ with host receptor *cone*-**1**, Figure S6: *K*_a_ (association constants) for *cone*-**1**·Ag^+^, Table S1: summary of crystal data for *cone*-**1**∙3MeOH∙H_2_O and *cone*-**1**∙2.5MeOH, Figure S7: Crystal structure of *cone*-**1**∙2.5MeOH, side view, Figure S8: Crystal structure of *cone*-**1**∙2.5MeOH, top view Figure S9: top view ball-and-stick *cone*-**1** ⊃ *n*-BuNH_3_^+^ complex, Figure S10: ball-and-stick *cone*-**1** ⊃ *n*-BuNH_3_^+^ complex, Figure S11: top view ball-and-stick *cone*-**1** ⊃ *t*-BuNH_3_^+^ complex, Figure S12: ball-and-stick *cone*-**1** ⊃ *t*-BuNH_3_^+^ complex, Table S2: calculated distances for selected parameters for the backbones of the host *cone*-**1** and complexes with Ag^+^ and *n*-BuNH_3_^+^ ions (Distance in Å).
