# Supplementary material for: A Hexahomotrioxacalix[3]arene-Based Ditopic Receptor for Alkylammonium Ions Controlled by Ag+ Ions
Source: Molecules. 2018 Feb 21;23(2):467. doi: 10.3390/molecules23020467 (PMC6017278; doi:10.3390/molecules23020467)
Supplement: Supplementary file 1 [file molecules-23-00467-s001.pdf]

Supporting information

Homooxacalix[3]arene-based Ditopic Receptor for Alkylammonium Ions Controlled by Ag^+ Ions

Xue-Kai Jiang [1], Yusuke Ikejiri [1], Chong Wu [1], Shofiur Rahman [2], Paris E. Georghiou [2], Xi Zeng [3], Mark R. J. Elsegood [4], Thomas G. Warwick [4], Carl Redshaw [5], Simon J. Teat [6], and Takehiko Yamato [1,]*

[1] Department of of Applied Chemistry, Faculty of Science and Engineering, Saga University, Honjo-machi 1, Saga 840-8502 Japan; jxk1215@163.com (X.K.J.); k1xshamgod@gmail.com (Y.I.); wuchong214@163.com (C.W.); yamatot@cc.saga-u.ac.jp (T.Y.)

[2] Department of Chemistry, Memorial University of Newfoundland, St. John's, Newfoundland and Labrador A1B 3X7, Canada; mdrahman71@yahoo.com (S.R.); parisg@mun.ca (P.E.G.)

[3] Key Laboratory of Macrocyclic and Supramolecular Chemistry of Guizhou Province, Guizhou University, Guiyang, Guizhou, 550025, China; zengxi1962@163.com(X.Z.)

[4] Chemistry Department, Loughborough University, Loughborough, LE11 3TU, UK; M.R.J.Elsegood@lboro.ac.uk (M.R.J.E.); Thomas.Warwick@nottingham.ac.uk (T.G.W.)

[5] School of Mathematics and Physical Sciences, The University of Hull, Cottingham Road, Hull, Yorkshire, HU6 7RX, UK; C.Redshaw@hull.ac.uk (C.R.)

[6] ALS, Berkeley Lab, 1 Cyclotron Road, Berkeley, CA 94720, USA; sjteat@lbl.gov (S.J.T.)

Table of contents

1. ^{1}H-NMR spectrum of the synthesized receptor *cone*-**1**……………………………Figure S1

2. ^{13}C-NMR spectrum of the synthesized receptor *cone*-**1**……………………………Figure S2

3. ^{1}H-NMR titration experiments for *cone*-**1** with *t*-$BuNH_3^+$ and Ag^+……………………Figure S3

4. ^{1}H-NMR titration experiments for *cone*-**1** with *n*-$BuNH_3^+$ and Ag^+……………………Figure S4

5. Molar ratio of Ag^+ with host receptor *cone*-**1**…………………………………………Figure S5

6. K_a (association constants) for *cone*-**1** ·Ag^+……………………………………………Figure S6

7. Summary of crystal data for *cone*-**1**·3MeOH·H_2O and *cone*-**1**·2.5MeOH..……………Table S1

8. Crystal structure of *cone*-**1**·2.5MeOH, side view...……………………….....………...Figure S7

9. Crystal structure of *cone*-**1**·2.5MeOH, top view...……….………………...………...Figure S8

10. Geometry-optimized Top view ball-and-stick *cone*-**1**⊃ *n*-$BuNH_3^+$ complex ………Figure S9

11. Geometry-optimized *cone*-**1** ⊃ *n*-$BuNH_3^+$ complex…………………………………Figure S10

12. Geometry-optimized Top view ball-and-stick *cone*-**1**⊃ *tert*-$BuNH_3^+$ complex……Figure S11

13. Geometry-optimized *cone*-**1** ⊃ *tert*-$BuNH_3^+$ complex………………………………Figure S12

14. Geometry-optimized *cone*-**1** ⊃ Ag^+ complex view from upper-rim…………………Figure S13

14. Calculated distances for selected parameters for the backbones of the host *cone*-**1** and complexes with Ag^+ and *n*-$BuNH_3^+$ ions (Distance in Å) ………………………………Table S2

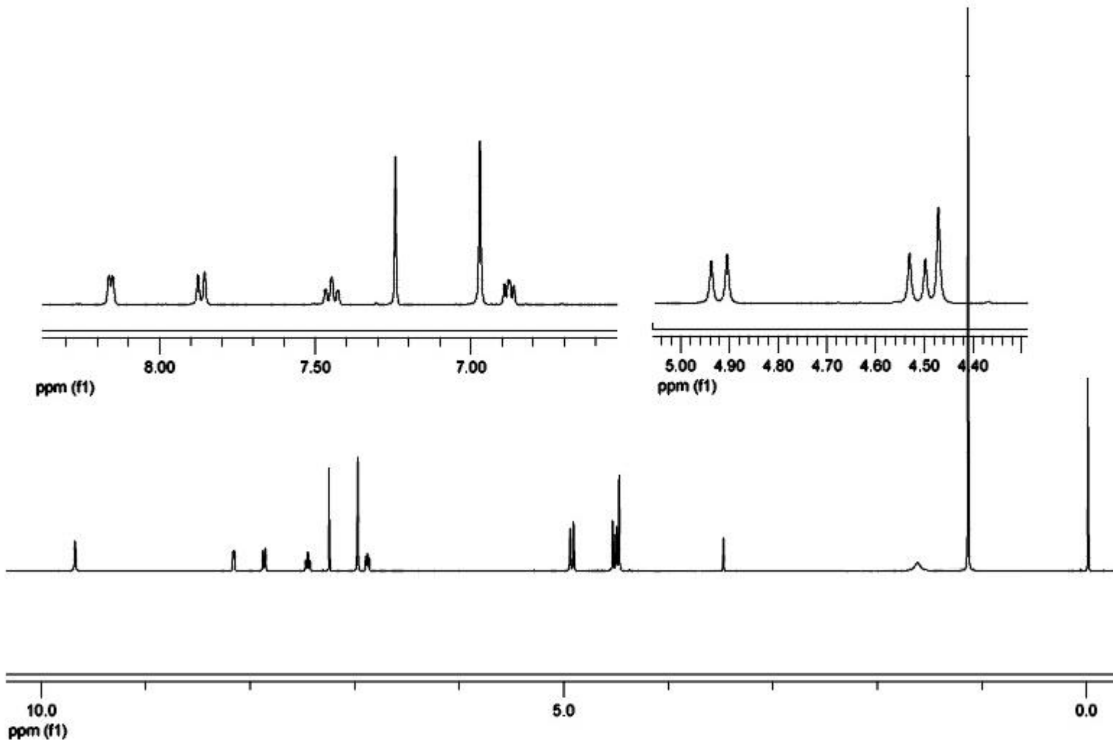

Figure S1. ^{1}H-NMR spectrum of receptor *cone*-**1** (300 MHz, $CDCl_3$, 293 K).

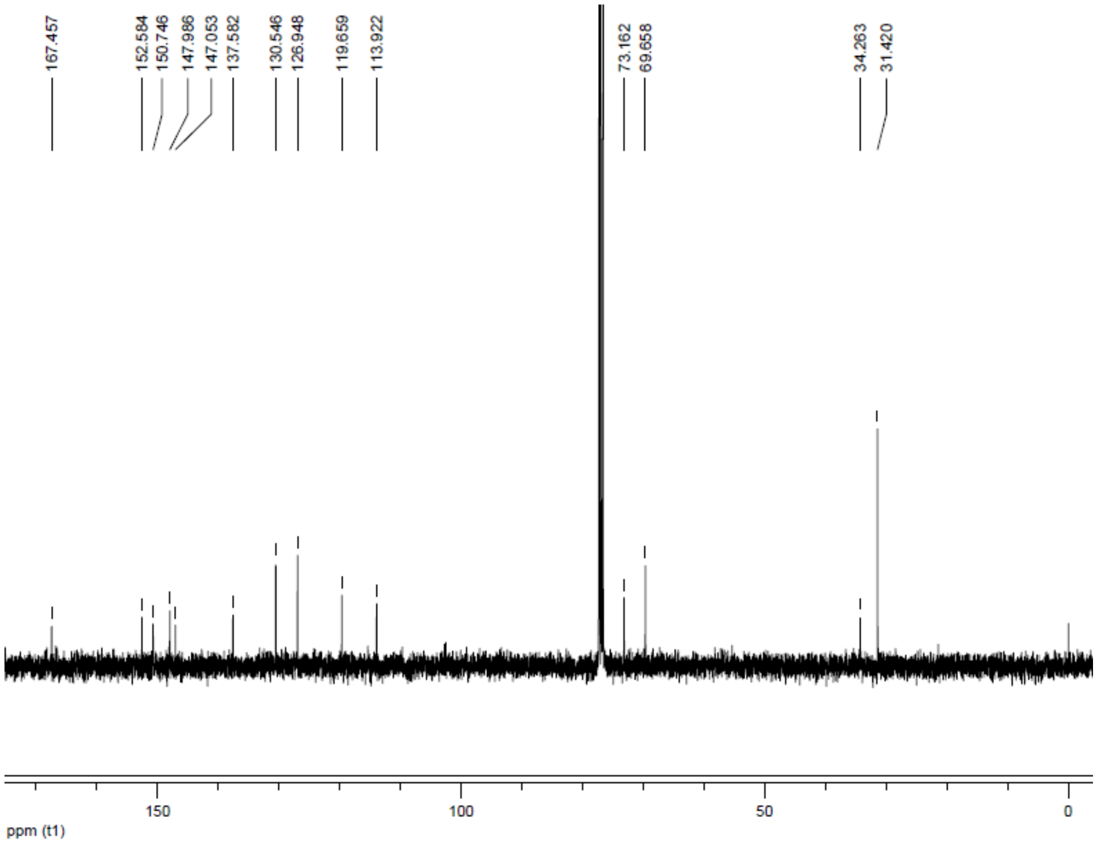

Figure S2. ^{13}C-NMR spectrum of receptor *cone*-**1** (100 MHz, $CDCl_3$, 293 K).

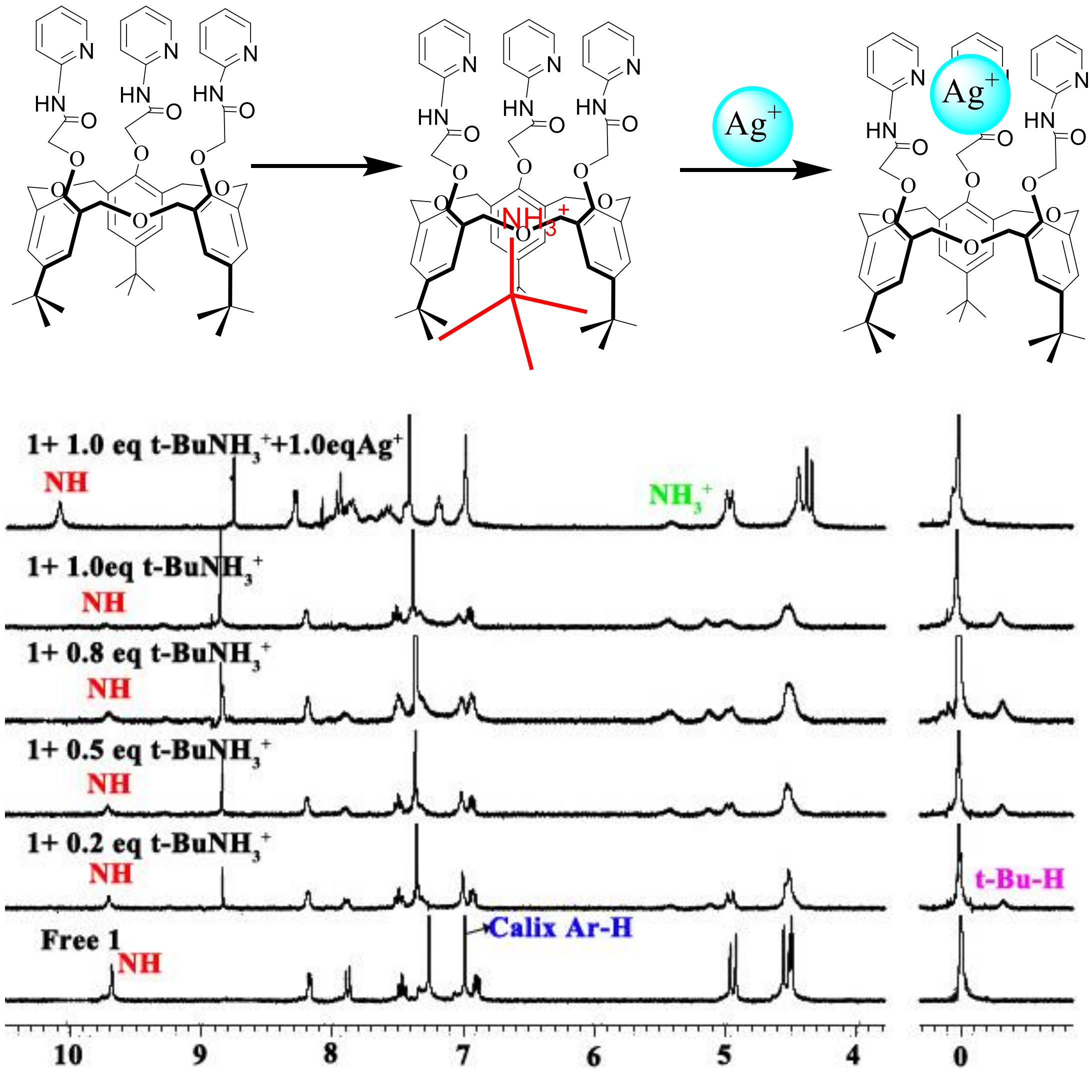

Figure S3. Partial ^{1}H-NMR spectral titration of receptor *cone*-**1**/*t*-BuNH$_3^+$ (H/G = 1:1); solvent: $CDCl_3/CD_3CN$ (10:1, v/v).

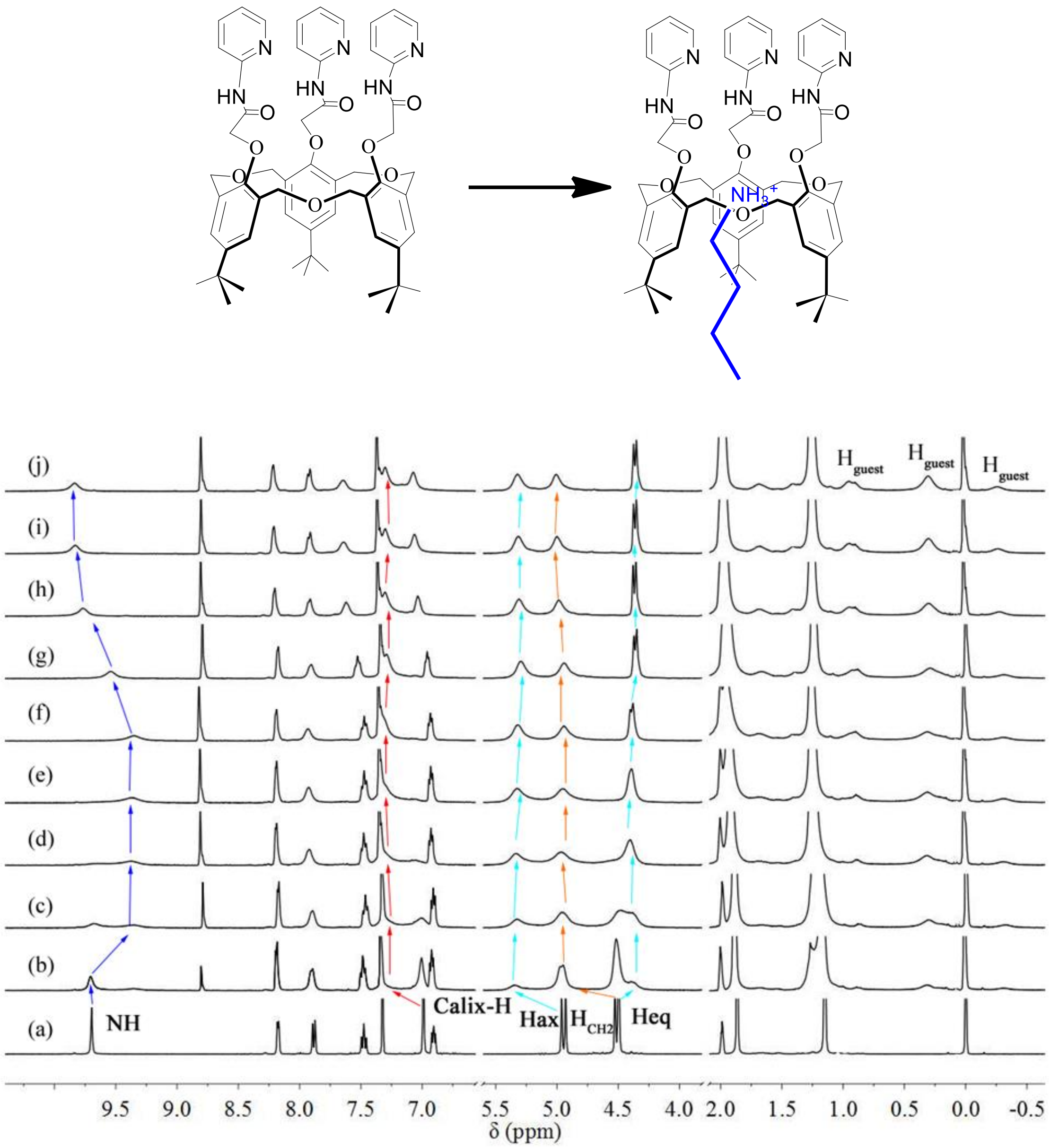

Figure S4. Partial ^{1}H-NMR spectral titration of receptor *cone*-**1**/guest complex (H/G = 1:1); a) free receptor *cone*-**1**; b) receptor *cone*-**1** ⊃ 0.2 equiv. of *n*-$BuNH_3^+$; c) receptor *cone*-**1** ⊃ 0.4 equiv. of *n*-$BuNH_3^+$; d) receptor *cone*-**1** ⊃ 0.6 equiv. of *n*-$BuNH_3^+$; e) receptor *cone*-**1** ⊃ 0.8 equiv. of *n*-$BuNH_3^+$; f) receptor *cone*-**1** ⊃ 1.0 equiv. of *n*-$BuNH_3^+$; g) receptor *cone*-**1** ⊃ 1.0 equiv. of *n*-$BuNH_3^+$ ⊃ 0.2 equiv. of Ag^+; h) receptor *cone*-**1** ⊃1.0 equiv. of *n*-$BuNH_3^+$ ⊃ 0.5 equiv. of Ag^+; i) receptor *cone*-**1** ⊃1.0 equiv. of *n*-$BuNH_3^+$ ⊃ 0.8 equiv. of Ag^+; j) receptor *cone*-**1** ⊃1.0 equiv.of *n*-$BuNH_3^+$ ⊃ 1.0 equiv. of Ag^+; solvent: $CDCl_3$/CD_3CN (10:1, v/v).

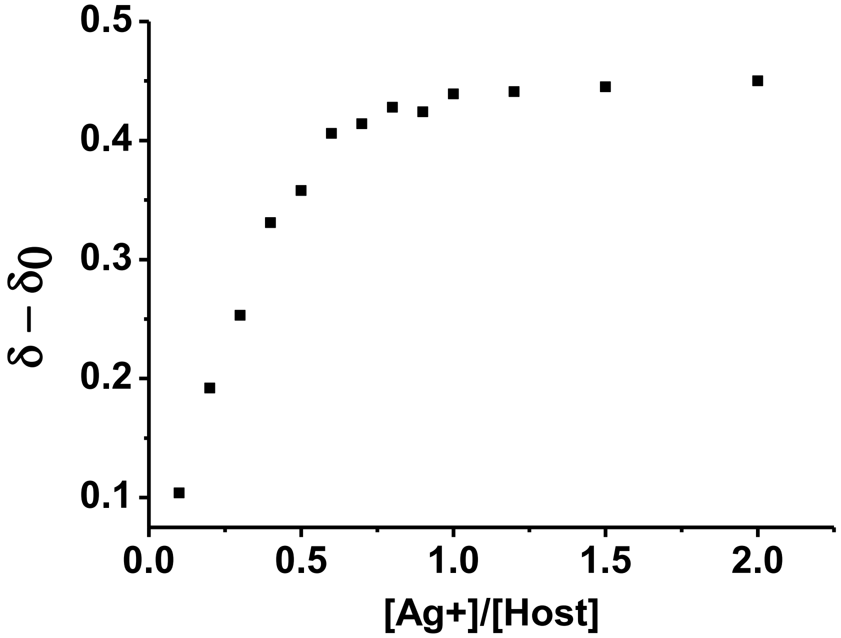

Figure S5. Molar ratio of Ag^+ with host receptor *cone*-**1**.

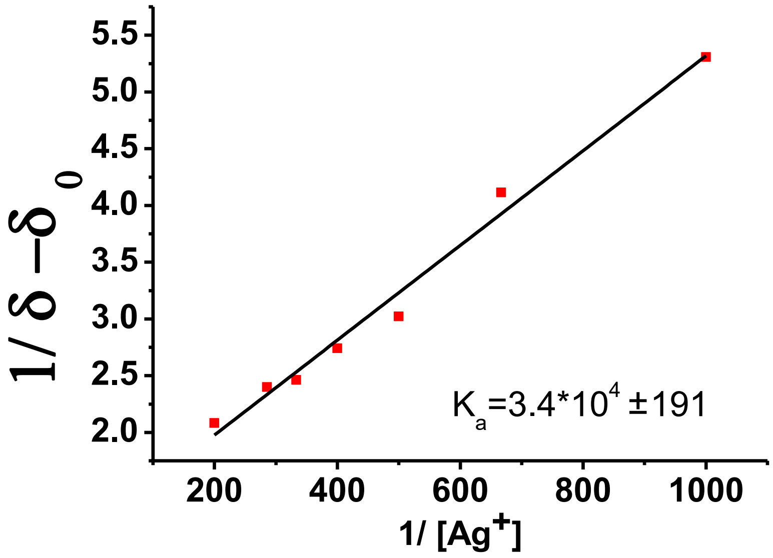

Figure S6. Bensei-Hilderbrand plot of receptor *cone*-**1** with varied concentrations of Ag^+ at 298 K. The associate constant (K_a) was calculated to be 3.4×10^4 M^{-1}.

X-ray crystallography

Table S1 Summary of crystal data for *cone*-**1**.[a,b]

Parameter	*cone*-**1**·3MeOH·H_2O	*cone*-**1**·2.5MeOH
Formula	$C_{57}H_{66}N_6O_9 \cdot 3(COH_4) \cdot H_2O$	$C_{57}H_{66}N_6O_9 \cdot 2.5(COH_4)$
Formula weight	1093.30	1059.26
Space group	*C*2/*c*	*C*2/*c*
a [Å]	26.1641(11)	26.308(2)
b [Å]	15.4995(6)	15.6159(14)
c [Å]	28.5153(11)	28.644(3)
β [°]	94.063(3)	94.1811(15)
Volume (Å^3)	11534.8(8)	11736.3(18)
Z	8	8
Wavelength [Å]	0.7085	0.71073
D(calc) [g.m^{-3}]	1.259	1.199
Temperature [K]	100(2)	150(2)
Measured reflns	113193	67896
Unique reflns	22109	17827
Obsd reflns [$I > 2\sigma(I)$]	16974	11159
Parameters	747	718
R_{int} [mm^{-1}]	0.074	0.053
R [I>2σ(I)][a]	0.067	0.059
wR [I>2σ(I)][b]	0.205	0.178
GOF on F^2	1.03	1.07

[a]Conventional R on F_{hkl}: $\Sigma ||Fo| - |Fc||/\sigma|Fo|$.

[b] Weighted R on $|F_{hkl}|^2$: $\Sigma [w(F_o^2 - F_c^2)^2]/ \ \Sigma [w(F_o^2)^2]^{1/2}$

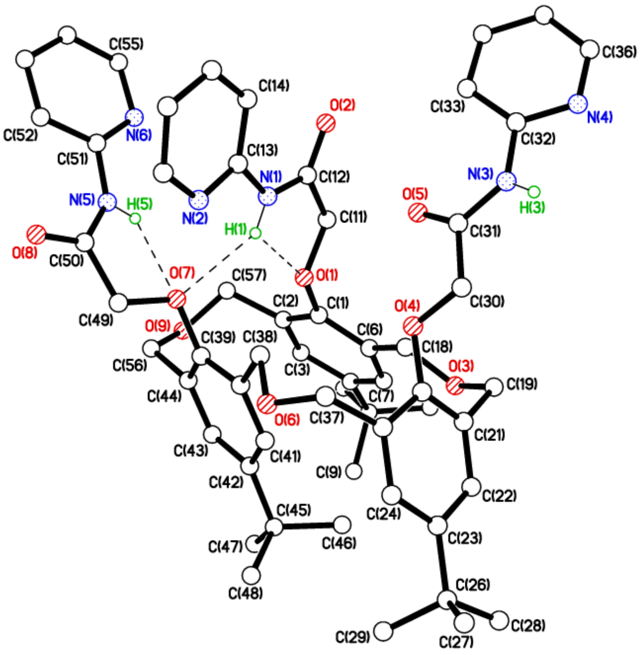

Figure S7. Crystal structure of *cone*-**1**·2.5MeOH; side view. MeOH of crystallization and H atoms not involved in H-bonding omitted for clarity.

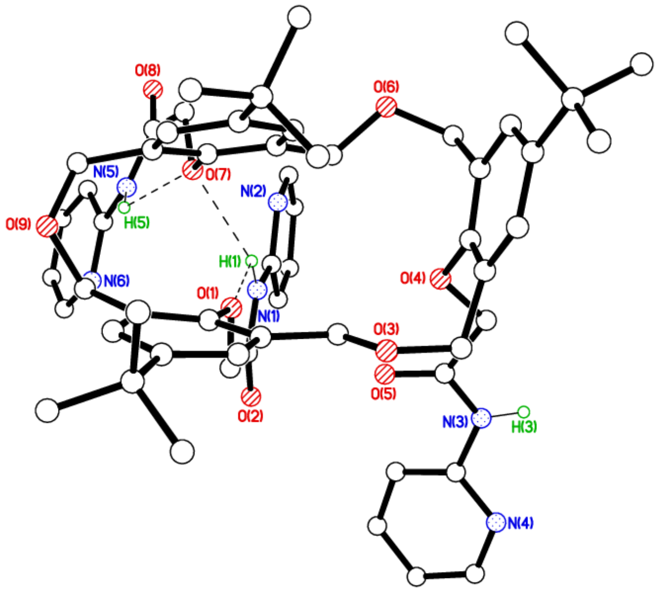

Figure S8. Crystal structure of *cone*-**1**·2.5MeOH; top view. MeOH of crystallization and H atoms not involved in H-bonding omitted for clarity.

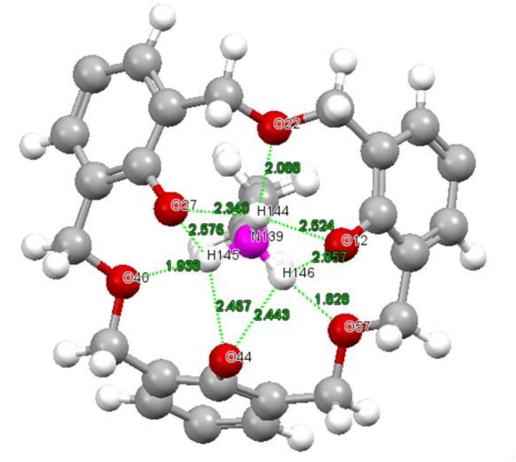

Number	Object1	Object2	Length
1	O12	H144	2.5242
2	O12	H146	2.8573
3	O27	H144	2.3399
4	O27	H145	2.5755
5	O44	H145	2.4675
6	O44	H146	2.4428
7	O22	H144	2.0878
8	O40	H145	1.9378
9	O57	H146	1.8279

Figure S9. Geometry-optimized (PBE0/LANL2DZ) structures (Ball-and-stick) of *cone*-**1** and as its complex with *n*-$BuNH_3^+$. Top: view of the *cone*-**1**⊃ *n*-$BuNH_3^+$ complex (*tert*-butyl groups at the upper rim and the pyridyl groups at the lower rim have been omitted for clarity). For bond distance values, see Table S2.

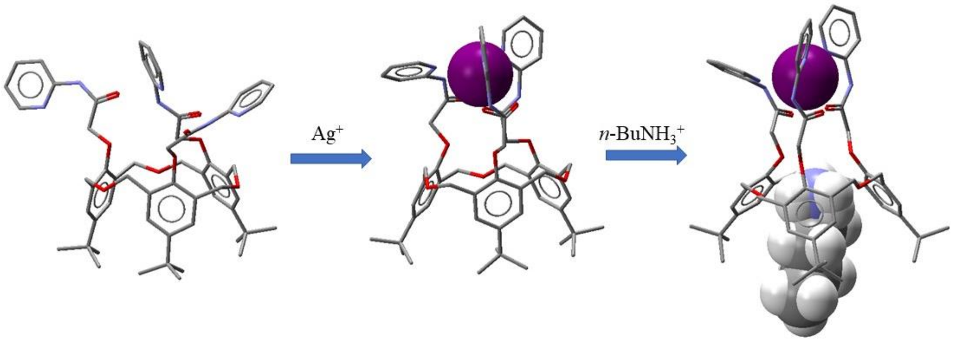

Figure S10. Geometry-optimized (PBE0/LANL2DZ) structures of *cone*-**1** and as its complex with *n*-$BuNH_3^+$. *Left*: The free *cone*-**1**. *Right*: 1:1 *cone*-**1**⊃ *n*-$BuNH_3^+$ complex. Colour code: carbon = drack grey, oxygen atom = red, nitrogen = blue and nitrogen (*n*-$BuNH_3^+$) = magenta. For bond distance values, see Table S2.

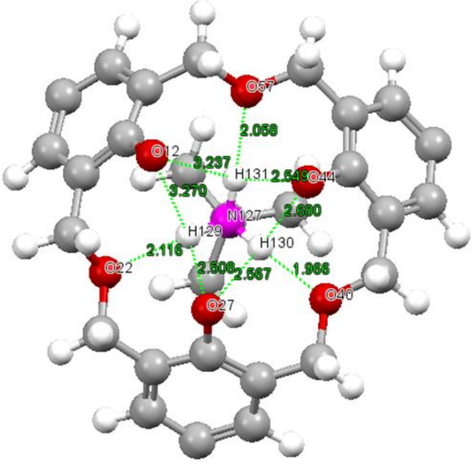

Number	Object1	Object2	Length
1	H129	O27	2.5084
2	H130	O44	2.6801
3	H131	O12	3.2373
4	H130	O40	1.9659
5	H129	O22	2.1162
6	H129	O12	3.2701
7	H131	O57	2.0577
8	H130	O27	2.5667
9	H131	O44	2.5494

Figure S11. Geometry-optimized (PBE0/LANL2DZ) structures (Ball-and-stick) of *cone*-**1** and as its complex with *tert*-BuNH$_3^+$. Top: view of the *cone*-**1** ⊃ *tert*-BuNH$_3^+$ complex (*tert*-butyl groups at the upper rim and the pyridyl groups at the lower rim have been omitted for clarity). For bond distance values, see Table S2.

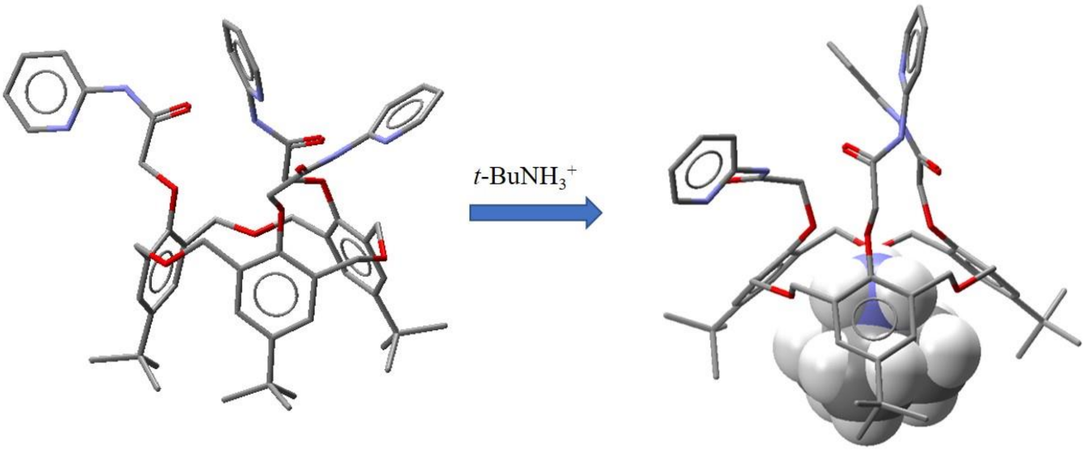

Figure S12. Geometry-optimized (PBE0/LANL2DZ) structures of *cone*-**1** and as its complex with *tert*-BuNH$_3^+$. *Left*: The free *cone*-**1**. *Right*: 1:1 *cone*-**1** ⊃ *tert*-BuNH$_3^+$ complex. Colour code: carbon = drack grey, oxygen atom = red, nitrogen = blue and nitrogen (*tert*-BuNH$_3^+$) = magenta. For bond distance values, see Table S2.

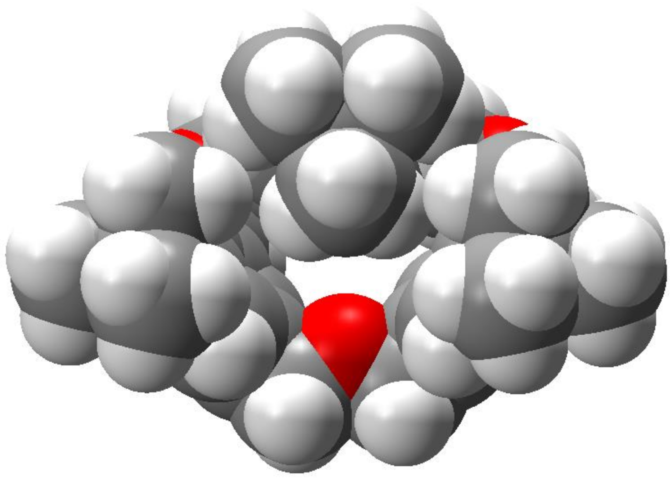

Figure S13. Geometry-optimized (PBE0/LANL2DZ) structures (space-filled) of *cone*-**1** and as its complex with Ag^+ from the wide-rim showing the compressed cavity entrance, due to the crowding of the three *tert*-butyl groups, in particular the one group which in pointing inwards towards an opposite aromatic ring.. The lower-rim functional groups of the *cone*-**1** Ag^+ complex have been have been omitted for clarity).

Table S2. The calculated distance for selected parameters for the backbones of the host *cone*-**1** and complexes with Ag^{+} and *n*-$BuNH_3^{+}$ ions in the gas phase at PBE0/LANL2DZ basis set (Distance in Å).

Parameter	*cone*-**1** Distance (Å)	*cone*-**1⊃Ag**$^{+}$ Distance (Å)	*n*-$BuNH_3^{+}$⊂ [*cone*-**1⊃Ag**$^{+}$] Distance (Å)	*cone*-**1**⊃ *n*-$BuNH_3^{+}$ Distance (Å)	*cone*-**1**⊃*tert*-$BuNH_3^{+}$ Distance (Å)
$N_6 – N_{48}$	5.0247	4.2956	4.130	7.100	7.427
$N_6 – N_{132}$	8.3503	3.8462	3.991	4.989	4.932
$N_{48} – N_{132}$	13.111	3.768	3.761	8.006	7.936
$O_9 – O_{35}$	4.9912	3.1731	3.224	3.373	3.330
$O_9 – O_{33}$	4.9425	3.1865	3.000	4.940	4.920
$O_{35} – O_{53}$	8.1903	6.2554	5.862	7.239	6.983
$O_{12} – O_{27}$	4.7736	4.2434	3.532	5.053	5.099
$O_{12} – O_{44}$	4.2481	4.7334	4.674	5.054	5.281
$O_{27} – O_{44}$	5.182	4.8133	4.139	4.995	5.108
$O_{22} – O_{40}$	6.2249	6.0583	5.061	3.836	3.842
$O_{22} – O_{57}$	5.636	5.9854	5.113	3.875	3.730
$O_{40} – O_{57}$	7.0824	7.2396	4.950	3.748	3.684
$N_6 –Ag_{156}$	–	2.4331	2.448	–	–
$N_{48} – Ag_{156}$	–	2.3271	2.364	–	–
$N_{132} – Ag_{156}$	–	2.4413	2.468	–	–
$O_9 –Ag_{156}$	–	2.3706	2.360	–	–
$O_{35} – Ag_{156}$	–	4.693	4.494	–	–
$O_{53} – Ag_{156}$	–	2.7364	2.617	–	–
$H_{144} – O_{22}$	–	–	2.382	2.524	3.237
$H_{144} – O_{27}$	–	–	2.234	2.088	2.116
$H_{145} – O_{27}$	–	–	2.233	2.340	2.508
$H_{145} – O_{40}$	–	–	2.678	2.576	2.567
$H_{145} – O_{44}$	–	–	1.906	1.938	1.966
$H_{145} – O_{12}$	–	–	3.069	2.468	2.549
$H_{145} – O_{44}$	–	–	2.896	2.857	3.270
$H_{146} – O_{57}$	–	–	3.209	2.443	2.680
$H_{146} – O_{57}$	–	–	1.818	1.828	2.058
